# Sequential deep learning image enhancement models improve diagnostic confidence, lesion detectability, and image reconstruction time in PET

**DOI:** 10.1186/s40658-024-00632-4

**Published:** 2024-03-15

**Authors:** Meghi Dedja, Abolfazl Mehranian, Kevin M. Bradley, Matthew D. Walker, Patrick A. Fielding, Scott D. Wollenweber, Robert Johnsen, Daniel R. McGowan

**Affiliations:** 1 Oxford University Hospitals, Oxford, UK; 2 GE HealthCare, Oxford, UK; 3 Cardiff University, Cardiff, UK; 4 GE HealthCare, Waukesha, WI USA; 5 University of Oxford, Oxford, UK

**Keywords:** Positron-emission tomography, Deep learning, Image reconstruction

## Abstract

**Background:**

Investigate the potential benefits of sequential deployment of two deep learning (DL) algorithms namely DL-Enhancement (DLE) and DL-based time-of-flight (ToF) (DLT). DLE aims to enhance the rapidly reconstructed ordered-subset-expectation-maximisation algorithm (OSEM) images towards block-sequential-regularised-expectation-maximisation (BSREM) images, whereas DLT aims to improve the quality of BSREM images reconstructed without ToF. As the algorithms differ in their purpose, sequential application may allow benefits from each to be combined. 20 FDG PET-CT scans were performed on a Discovery 710 (D710) and 20 on Discovery MI (DMI; both GE HealthCare). PET data was reconstructed using five combinations of algorithms:1. ToF-BSREM, 2. ToF-OSEM + DLE, 3. OSEM + DLE + DLT, 4. ToF-OSEM + DLE + DLT, 5. ToF-BSREM + DLT. To assess image noise, 30 mm-diameter spherical VOIs were drawn in both lung and liver to measure standard deviation of voxels within the volume. In a blind clinical reading, two experienced readers rated the images on a five-point Likert scale based on lesion detectability, diagnostic confidence, and image quality.

**Results:**

Applying DLE + DLT reduced noise whilst improving lesion detectability, diagnostic confidence, and image reconstruction time. ToF-OSEM + DLE + DLT reconstructions demonstrated an increase in lesion SUV_max_ of 28 ± 14% (average ± standard deviation) and 11 ± 5% for data acquired on the D710 and DMI, respectively. The same reconstruction scored highest in clinical readings for both lesion detectability and diagnostic confidence for D710.

**Conclusions:**

The combination of DLE and DLT increased diagnostic confidence and lesion detectability compared to ToF-BSREM images. As DLE + DLT used input OSEM images, and because DL inferencing was fast, there was a significant decrease in overall reconstruction time. This could have applications to total body PET.

**Supplementary Information:**

The online version contains supplementary material available at 10.1186/s40658-024-00632-4.

## Introduction

Positron Emission Tomography (PET) is an established non-invasive image modality that allows for visualisation of metabolic processes in the body, particularly in the settings of oncology [1], cardiology [2], and neurology [3]. Although PET is renowned for its sensitivity, spatial resolution is relatively low, and images can be limited by noise [4]. Many improvements have been made to PET instrumentation to address these limitations; these include hardware-based solutions such as optimisations of scintillators, photodetectors, extending the axial field-of-view, and implementation of time-of-flight technology (ToF). ToF detectors measure the annihilation photon arrival times with sufficient precision to constrain the location of the annihilation along the line of response, which leads to an increased signal-to-noise ratio in the resulting PET image [5–10].

Software-based solutions to improve PET image quality include advances in system modelling and in reconstruction algorithms. Iterative reconstruction methods, such as the ordered subset expectation maximisation (OSEM) algorithm [11], are frequently used for PET reconstructions. Since image noise increases with the number of iterations, OSEM is often stopped before approaching convergence with additional filters then applied to suppress noise [12]. In contrast, the block sequential regularised expectation maximisation (BSREM) algorithm inherently controls noise allowing for preservation of image quality at effective convergence [13]. BSREM uses a regularisation (or penalty) factor that steers image updates away from noisier solutions. Bayesian reconstructions such as BSREM have gained widespread acceptance but are more computationally expensive to date and have not supplanted OSEM as an industry standard, being unavailable from some vendors. The substantial hardware and software improvements that have been progressively realised since the advent of PET- Computed Tomography (CT) have combined to give improved diagnostic confidence and lesion detectability [14, 15], while facilitating shorter imaging times and reductions in injected activities [16].

In recent years, Machine Learning (ML) applications in PET have produced promising results. ML has been applied in multiple aspects of PET, including image reconstruction, image acquisition, attenuation/scatter correction and quantitative imaging. Applications frequently use deep learning (DL) network structures such as convolutional neural networks (CNNs), generative adversarial networks (GANs) and U-nets. While improved time resolution can be achieved with recent developments of ToF detectors, DL has been used to improve image quality of non-ToF scanners by training algorithms to reconstruct images with ToF-like benefits without ToF information [17, 18]. Assessments of image quality from the DL-based time-of-flight (DLT) algorithm were similar or superior to the images reconstructed using the native ToF information [17]. Multiple studies are providing support towards the deployment of DL to estimate the interaction energy, position, and arrival time of incident photons. However, this comes with a variety of difficulties such as achieving the required training data through extensive experimental measurements.

Given the ability of solving complex inverse problems, DL algorithms are suitable for various image reconstruction or image processing applications in PET. There are different approaches to solve the complex problems: one approach is to use DL to generate standard quality images from low-quality images. In this approach, algorithms are trained to improve images acquired from shorter acquisitions or lower injected activity scans, which are enhanced towards images of standard quality. A Deep Learning Enhancement (DLE) algorithm for ^18^F-FDG PET scans used deep neural networks to enhance rapidly reconstructed OSEM images to make them BSREM-like. Using the DLE algorithm, the computational reconstruction time was significantly reduced, and the image quality was maintained [19]. Another approach is to use DL to reconstruct PET images from sinograms [20, 21]. Other approaches, such as ‘hybrid domain learning’ rely on both ML approaches as well as analytical solutions to reach an optimal image [22].

The aim of the current study is to investigate the potential benefits of sequential deployment of two deep learning (DL) algorithms namely DL Enhancement (DLE) and DL-based time-of-flight (DLT).

Such (mis-) use brings risks of unexpected or undesired outcomes, but with potential for significant benefits in terms of image quality; we aimed to explore both aspects. DLE aims to enhance the rapidly reconstructed OSEM images towards BSREM images, whereas DLT aims to improve the quality of BSREM images reconstructed without ToF towards ToF-BSREM. As the algorithms differ in their purpose, the sequential application may allow the benefits from each to be combined.

## Materials and methods

### Reconstructions

PET raw data (i.e., sinograms) were reconstructed using five combinations of algorithms as shown in Table 1.

**Table 1:** Deep learning algorithm combinations explored in this study with brief descriptions

Reconstruction number	Reconstruction and DL name	Comment
1	ToF-BSREM	Gold standard in the current study, widespread clinical use.
2	ToF-OSEM + DLE	Computationally fast. Evaluated previously [21]
3	OSEM + DLE + DLT	Computationally fast, Use of DLE is outside bounds of DL training data.
4	ToF-OSEM + DLE + DLT	Computationally fast. Use of DLT is outside bounds of DL training data.
5	ToF-BSREM + DLT	Use is outside bounds of DLT training data.

Reconstruction one (ToF-BSREM) is the gold standard, all subsequent reconstructions were compared against it. ToF-OSEM is the expected input for DLE, as used in reconstruction two (ToF-OSEM + DLE) and previously evaluated [19]. Reconstructions three (OSEM + DLE + DLT), four (ToF-OSEM + DLE + DLT) and five (ToF-BSREM + DLT) apply the DL algorithms beyond their intended use. In these cases, the input image being provided to the DL algorithm potentially has characteristics or features that are outside the boundaries of the training data used in creation of the DL model.

DLE is a 3D residual convolutional coder-decoder (U-Net) network developed and implemented in Pytorch. The model was trained in supervised sessions, where it mapped low-contrast high-noise OSEM PET images to low noise high contrast BSREM images. In the supervised sessions, the output OSEM + DLE was compared to a target patch BSREM and based on the result of the smooth L1 loss function the trainable parameters were updated. The DLE architecture is composed of convolutional layers (using 3 × 3 × 3 kernels), batch normalisation (BN), 3D max pooling layers and tri-linear up-sampling layers, skip and residual connections and leaky rectified linear unit (ReLU) activation functions. DLE used 510 [^18^F]-FDG PET/CT scans from six sites equipped with D710 and DMI, of these scans, *n* = 480 were used for training, *n* = 15 for validation and *n* = 25 for testing. The OSEM reconstructions were of matrix size 256 × 256, field-of-view 700 mm, voxel size 2.7 × 2.7 × (2.8 or 3.7) mm^3^ and 2 iterations, 34 and 24 subsets for Discovery MI and 710 scanners, respectively, with PSF and standard z-filter [19].

DLT is similar to DLE’s network that is a 3D residual U-Net developed and implemented in Pytorch. The model was trained in supervised sessions, where DLT compared the predicted ToF-BSREM images to target ToF-BSREM using the MSE loss function. For DLT, a total of 273 [^18^F]-FDG PET/CT scans were used from six sites equipped with DMI scanners only, split into training (*n* = 208), validation (*n* = 15), and testing (*n* = 50) sets. The matrix size of each reconstructed image was of 256 × 256 and field-of-view of 700 mm (x–y pixel size: 2.73 mm, slice thickness: 2.79 mm) [17].

The manufacturer’s Bayesian penalised-likelihood reconstruction algorithm was used to reconstruct all BSREM images with a fixed regularization factor, β = 400 [13]. All OSEM reconstructions used two iterations with 32 (D710) or 34 (DMI) subsets, no in-plane (x-y) post filter, and with the manufacturer’s standard z-filter which is the anticipated (trained) input for the DLE model. Out of the three DLT models available (low, medium, high) with names that describe the strength with which the models were trained to transform non-ToF BSREM images to their target ToF BSREM, the current study used ‘high’ consistently. This was based on previous reader preference [17], and to provide greater changes to the images thus increasing the likelihood of detectable differences in the current study while limiting the number of variables under examination.

### Patient selection

The study made use of 40 whole-body ^18^F-FDG PET-CT scans. 20 scans were performed sequentially on a Discovery 710 and 20 on 25 cm axial field of view Discovery MI (both GE HealthCare). All scans were free breathing, and ungated. For each patient, a whole-body helical CT was performed for PET attenuation correction using 100–120 kVp, 150–200 mAs. For the DMI subjects, the range in activity was (mean ± std MBq) (391.4 ± 99.0 MBq), the patient size range was BMI (26.9 ± 5.6 kg/m^2^), FDG uptake time range was (80.1 ± 21.2 min) and the acquisition time per bed position was two minutes per bed position. For the D710 subjects, the range in activity was (309.0 ± 77.6 MBq). The patient size range was (27.2 ± 6.8 kg/m^2^), the FDG uptake time range was (89.7 ± 7.9 min) and all scans were three minutes per bed position.

### Clinical and quantitative evaluation

Two experienced radiologists, reader 1 (K.M.B. 20 years board certified in clinical radiology and nuclear medicine) and reader 2 (P.A.F. 19 years board certified in clinical radiology and nuclear medicine), blinded to method of reconstructions, rated the images on a Likert scale (5 best) based on diagnostic confidence, lesion detectability and image quality. The Likert scale used was 0 (non-diagnostic), 1 (poor,), 2 (satisfactory), 3 (good), 4 (very good), and 5 (excellent) as in previous work [17]. Inter-reader agreement was determined using Intraclass Correlation Coefficient (ICC) (two-way random effects model) carried out in SPSS 29. To check for differences across groups a Friedman test was carried out. When significant differences were found, Wilcoxon signed-ranks test with Bonferroni post-hoc were performed for pair-wise comparisons with a significance threshold of *p* < 0.05.

Radiologist K.M.B identified lesions that were subtle and/or small and recorded the SUV_max_ of these lesions. To assess noise, the standard deviation between voxels within a 30 mm spherical VOI was calculated using VOIs placed in normal lung and liver. Group-wise differences were calculated using the Kruskal-Wallis test. When significant differences were found, Wilcoxon signed-ranks test with Bonferroni post-hoc were performed for pair-wise comparisons with a significance threshold of *p* < 0.05. Friedman and Wilcoxon-signed ranks tests were carried out in Python 3.11.

## Results

**Fig. 1:**
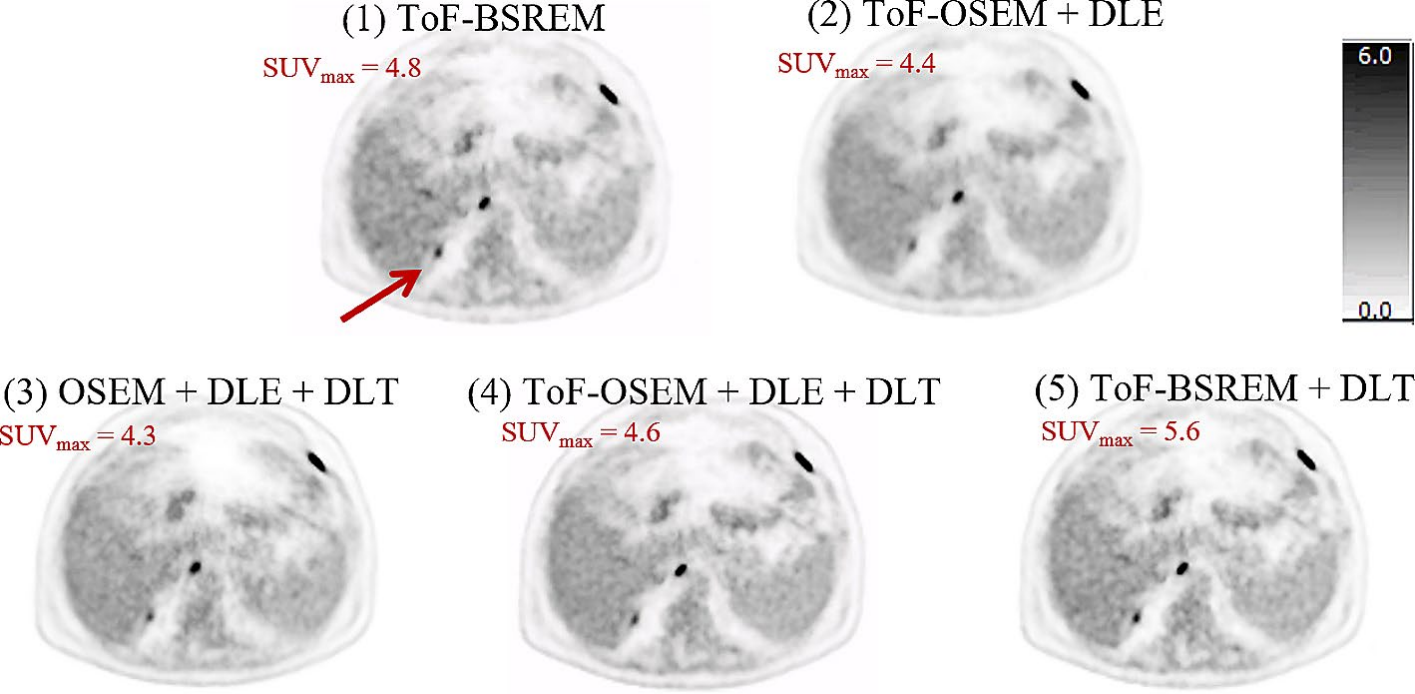
Deep Learning Enhancement (DLE) + Deep Learning Time-of-flight (DLT) applications to a test subject with BMI 19.4 kg/m^2^, with an injected activity of 229 MBq FDG, scanned on a D710 PET-CT scanner. The subject is a male patient, staging scan for relapsed, high grade, transformed, follicular, non-Hodgkin’s lymphoma, with nodal and peritoneal disease. Axial PET images and the SUV_max_ of a tiny peritoneal nodule posterior to the right lobe of the liver (red arrow) are demonstrated. All images use an SUV scale of 0–6

**Fig. 2:**
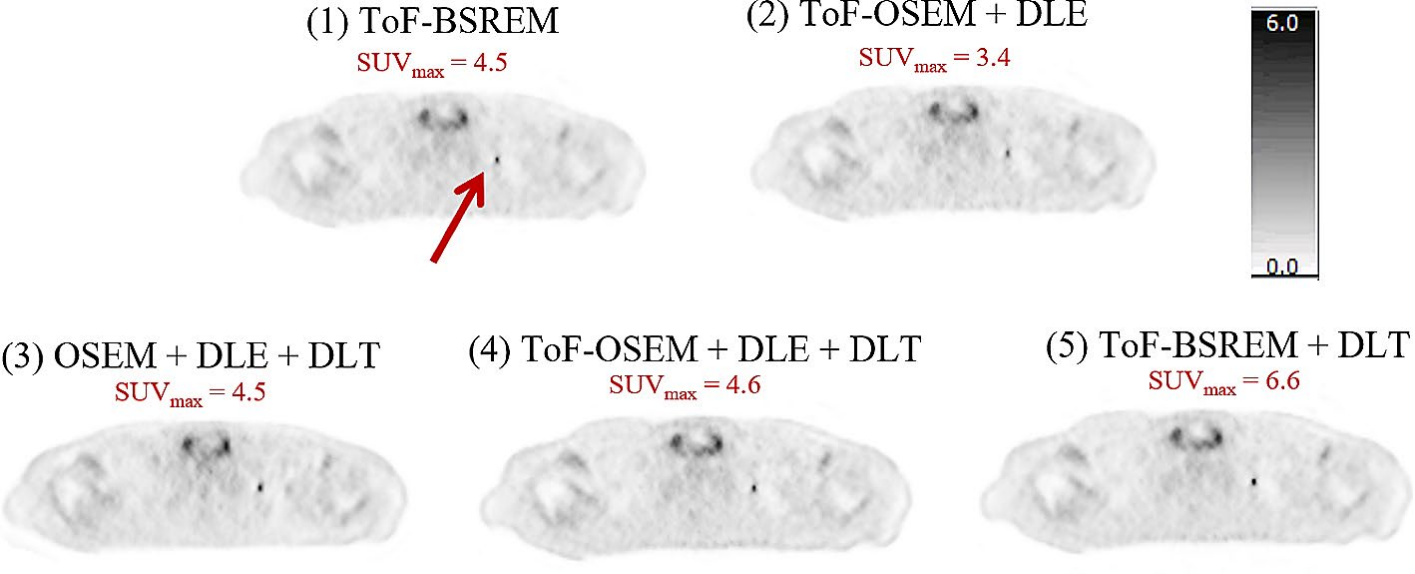
Deep Learning Enhancement (DLE) + Deep Learning Time-of-flight (DLT) applications to a test subject with BMI 27.4 kg/m^2^, with an injected activity of 419.0 MBq FDG, scanned on a DMI PET-CT. The subject is a female patient, staging scan for breast carcinoma, with left axillary and a solitary, approximately 7 × 4 mm left supraclavicular node. Axial PET images and the SUV_max_ of the left supraclavicular fossa node (red arrow) are demonstrated. All images use an SUV scale of 0–6

**Fig. 3:**
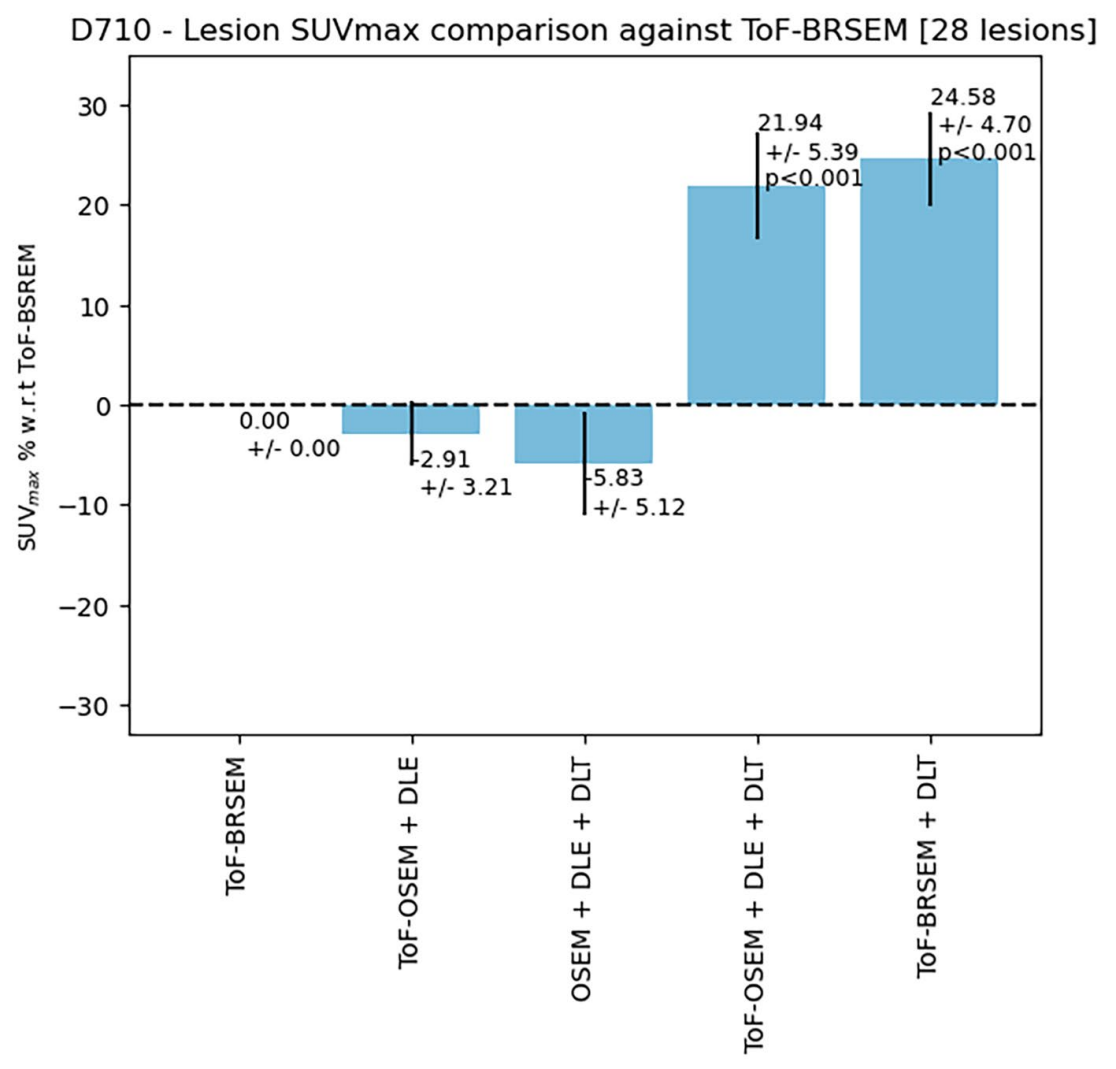
Quantitative performance of five algorithms on lesion SUVmax scanned on a D710 PET-CT scanner. All *p*-values are calculated using the Wilcoxon signed-rank test and Bonferroni-corrected. *P*-values are not displayed if larger than 0.05. All lesions chosen were extreme cases where lesions were small (sub-cm)

**Fig. 4:**
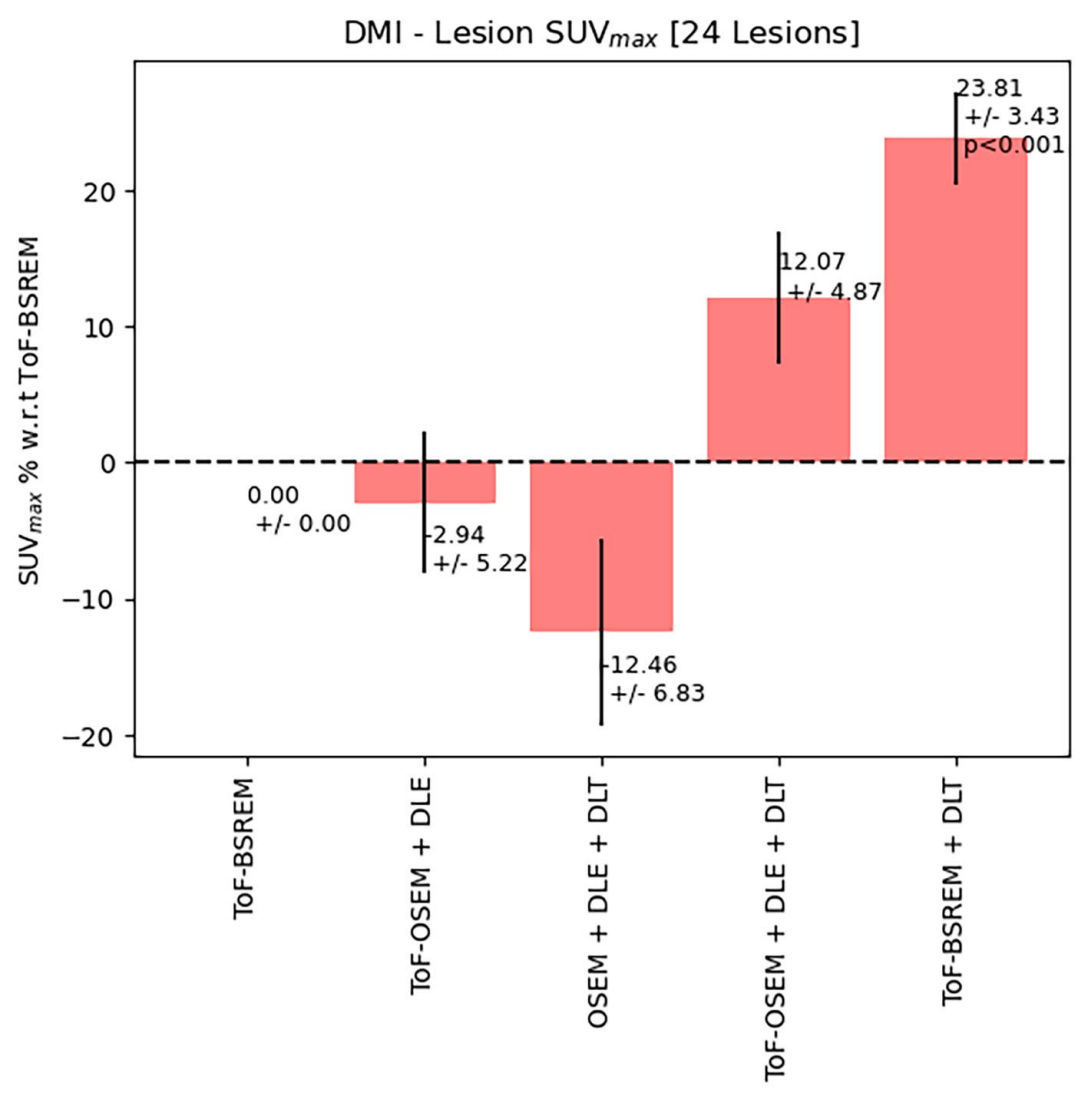
Quantitative performance of five algorithms on lesion SUVmax scanned on a DMI PET-CT scanner. All *p*-values are calculated using the Wilcoxon signed-rank test and Bonferroni-corrected. *P*-values are not displayed if larger than 0.05. All lesions chosen were extreme cases where lesions were small (sub-cm)

Figures 1 and 2 display two example performances of the five reconstructions on two subjects. In Fig. 1, the images show that when DLE is applied the liver appears smoother, whereas when DLT is applied the lesion is more conspicuous, though the image may suffer from higher noise. In reconstruction four, where DLE + DLT is applied sequentially to ToF-OSEM, improvements due to both DL algorithms are visible. In Fig. 2, the lesion indicated by the arrow is small and of low uptake. The lesion is most visible in reconstructions one, four and five. This visibility is also reflected in the SUV_max_ values, where reconstructions five and four were highest and second highest respectively. The results for all lesion SUV_max_ values are summarised in Figs. 3 and 4.

Figures 3 and 4 display the quantitative performance of reconstruction methods 2–5 for SUV_max_ relative to method one as a percentage difference. SUV_max_ values for BSREM reconstructions were included in both Figures to display the impact of ToF-information on SUV_max_. Figure 3 shows the results obtained for 28 challenging lesions from the D710 scans. Figure 4 shows the results obtained for 24 challenging lesions from the DMI scans. Error bars represent the standard error of the mean and *p*-values, calculated using the Wilcoxon-signed ranks test and corrected for multiple comparisons, are displayed if smaller than the significance level 0.05. Figure 3 shows that in the D710 data set, there is no significant difference between method one and methods two and three. A statistically significant increase in SUV_max_ is found in reconstructions four and five compared to one. Similarly, to the D710 data set, reconstruction five outperforms all other methods.

**Fig. 5:**
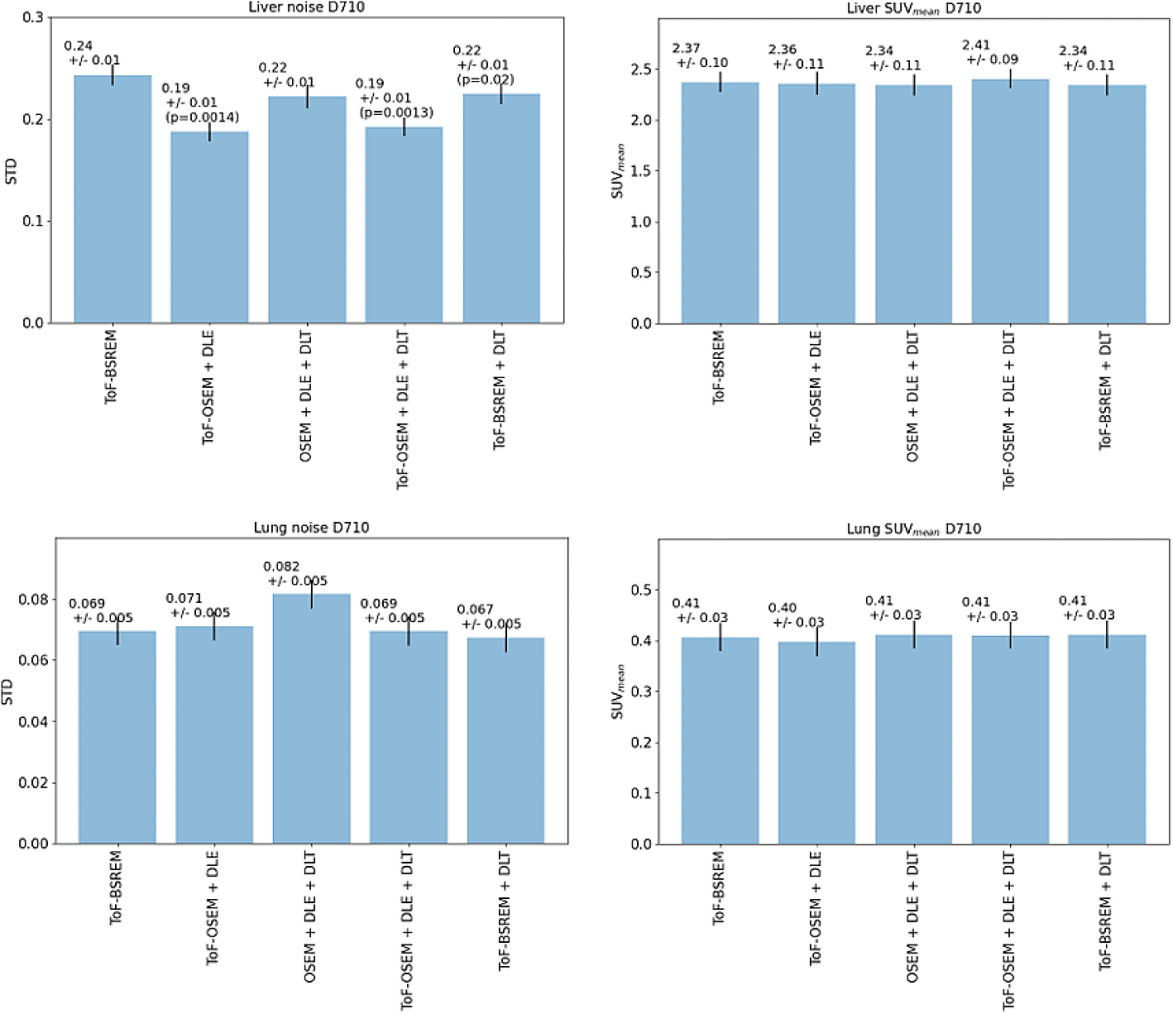
Quantitative performance of five reconstructions of DLE/DLT combinations. Tested by drawing a 30 mm spherical VOI in both the lung and liver and measuring standard deviation between voxels and SUVmean for 20 patients scanned on a GE HealthCare Discovery 710 scanner

**Fig. 6:**
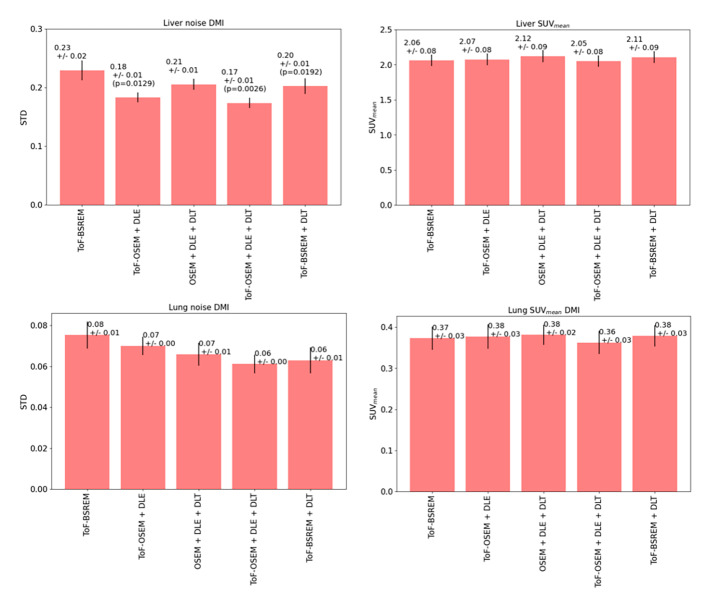
Quantitative performance of five reconstructions of DLE/DLT combinations. Tested by drawing a 30 mm spherical VOI in both the lung and liver and measuring standard deviation between voxels and SUVmean for 20 patients scanned on the GE DMI 10 scanner

Figures 5 and 6 displays the quantitative performance of reconstructions 1–5 for noise and SUV_mean_ in the lung and liver. Error bars represent the standard error of the mean, *p*-values calculated using the Wilcoxon-signed ranks test, and corrected for multiple comparisons, are displayed if smaller than the significance level 0.05. Figure 5 shows the results obtained from the D710 data set whereas Fig. 6 shows the results obtained from the DMI data set. All reconstructions in the DMI and D710 dataset gave similar SUV_mean_ values for the uniform liver and lung region (Kruskal-Wallis *p* > 0.05). In both D710 data and DMI data, noise in the liver was significantly reduced when DLE and DLT was applied sequentially. No significant difference was found in lung noise in the DMI data set (Kruskal-Wallis *p* > 0.05).

### Clinical reading

Tables 2 and 3 show the scores for reconstruction methods 1–5 Table 2 shows the scores for the D710 data set and Table 3 shows the scores the DMI data set. No images were scored as non-diagnostic. The values in bold are the highest score for each metric. Table 2 shows a preference for reconstructions four and five in lesion detectability and diagnostic confidence. Whereas reconstructions two and four were preferred for image quality. A similar pattern is displayed in the DMI dataset in Table 3 where there is a preference for reconstructions four and five in lesion detectability and diagnostic confidence and a preference for reconstruction two and four for image quality. Both tables show an overall preference for reconstruction four which deploys DLE + DLT on ToF-OSEM.

**Table 2:** Clinical image scoring from two readers of 20 whole-body FDG scans acquired on the D710 scanner. *p*-values were calculated using the Wilcoxon signed-rank test. The values in bold represent the best (highest) score for each metric

Reconstruction	Lesion detectability	Diagnostic confidence	Image quality
ToF-BSREM	3.50 ± 0.07	3.35 ± 0.08	2.50 ± 0.10
ToF-OSEM + DLE	3.27 ± 0.10 (*p* = 0.19)	3.33 ± 0.10 (*p* = 1.00)	**3.98 ± 0.13** **(** ***p*** **= 0.001)**
OSEM + DLE + DLT	2.88 ± 0.17 (*p* = 0.03)	2.77 ± 0.16 (*p* = 0.04)	2.95 ± 0.11 (*p* = 0.04)
ToF-OSEM + DLE + DLT	**4.42 ± 0.11** **(** ***p*** **< 0.001)**	**4.45 ± 0.11** **(** *p* **= 0.001)**	3.88 ± 0.10 (*p* = 0.001)
ToF-BSREM + DLT	4.28 ± 0.11 (*p* = 0.001)	4.15 ± 0.10 (*p* = 0.001)	2.88 ± 0.08 (*p* = 0.009)
ICC	0.56	0.57	0.65

**Table 3:** Clinical image scoring from two readers of 20 whole-body FDG scans acquired on a DMI scanner. *p*-values were calculated using the Wilcoxon signed-rank test. The values in bold represent the best (highest) score for each metric

Reconstruction	Lesion detectability	Diagnostic confidence	Image quality
ToF-BSREM	3.62 ± 0.11	3.55 ± 0.12	2.77 ± 0.13
ToF-OSEM + DLE	3.48 ± 0.09 (*p* = 1.00)	3.52 ± 0.09 (*p* = 1.00)	3.65 ± 0.12 (*p* = 0.005)
OSEM + DLE + DLT	2.55 ± 0.13 (*p* = 0.002)	2.50 ± 0.12 (*p* = 0.001)	2.85 ± 0.15 (*p* = 1.00)
ToF-OSEM + DLE + DLT	4.05 ± 0.13 (*p* = 0.27)	4.10 ± 0.11 (*p* = 0.05)	**4.03 ± 0.10** **(** *p* **= 0.001)**
ToF-BSREM + DLT	**4.15 ± 0.07** **(** *p* **= 0.004)**	**4.12 ± 0.07** **(** *p* **= 0.004)**	3.40 ± 0.12 (*p* = 0.001)
ICC	0.41	0.33	0.51

## Discussion

The present study investigated the potential benefits of sequential deployment of two deep learning (DL) algorithms: DLE and DLT. Five reconstruction algorithms were tested on 40 FDG scans from two different scanners. Reconstruction four (ToF-OSEM + DLE + DLT) was the optimal combination out of the five reconstructions. It produced larger decrease in liver noise, significant increase in SUV_max_ and it was the overall preference in the clinical reading.

Reconstruction four (ToF-OSEM + DLE + DLT) and reconstruction five (ToF-BSREM + DLT) demonstrated a larger increase in lesion SUV_max_ relative to the gold standard reconstruction one (ToF-BSREM). Reconstruction five demonstrated the largest increase in SUV_max,_ particularly in the DMI data. This gain in SUV_max_ was reflected in the DMI clinical scoring, where reconstruction five and four scored highest and second highest for both lesion detectability and diagnostic confidence with small differences between their respective average reader scores (0.1 and 0.02 respectively). However, reconstruction five images were reported to suffer from noise, thus lowering the image quality score to third lowest. Regarding image quality, reconstruction four scored the highest. In the D710 data, where the gain in SUV_max_ between reconstructions four and five are similar, there is a preference for reconstruction four in both lesion detectability and diagnostic confidence due to less noise. Reconstruction two scored highest for image quality with reconstruction four being a close second, both reconstructions include DLE, further showcasing the algorithm’s denoising abilities. Furthermore, both DL algorithms have been trained on DMI data which has better ToF timing resolution, therefore, the benefits of applying DL may be higher for D710 input image data.

In addition to improving the lesion detectability, diagnostic confidence, and image quality, using DLE has the advantage of reducing the computational burden of the reconstructions. For example, on the DMI scanner, reconstruction time of BSREM and OSEM algorithms are 3.1 and 1.1 min per bed position respectively, as previously reported [19].

Whereas the addition of DLE for processing a whole-body scan is only ∼ 5s [19]. Therefore, DLE reconstructed images take a third of the reconstruction time and are comparable to BSREM images. This large saving in reconstruction time could significantly affect the feasibility of algorithm deployment and routine clinical use, especially in the case of larger field of view cameras where reconstruction times need to keep pace with higher patient throughput. The reconstruction times for the patients from each type of reconstruction, reconstructed using a GE research system, is provided in the supplementary Table S1.

There were some necessary limitations to this study, which aimed not for clinical validation but for rapid exploration of algorithm performance. The number of readers was only two, and there were only a moderate number of exams included in the study (40). Five combinations of algorithms were assessed, resulting in 400 whole body FDG PET/CT reviews in total. We considered this to be reasonable to meet the study aims but it is not exhaustive. Other image reconstruction and DL combinations are possible, we noted that the successive application of DLT led to noticeable image artifacts. No reconstruction artefacts were noted by the radiologists within any of the images presented in this paper, where DLT and DLE were combined. We sequentially applied DLT to ToF-BSREM images six times, results for lung and liver noise as well as lesion SUV_max_ can be found in supplementary Figures S1 and S2. Example images can be found in supplementary Figure S3 where image artefacts are visible following multiple applications of DLT.

The input images were of standard injected activity (or acquisition time), and we did not assess performance in the case of lower quality input images. Furthermore, only two PET-CT scanner models were included in the assessment and while this gives an indication that the results may be generalisable, similar evaluations using additional scanner models would give greater assurance in this regard. Images were assessed in terms of several quantitative metrics and scored by two readers, but the study could not assess performance for lesion detection or the rates of false positives which would be important considerations prior to any clinical use.

We recognise that in this study there were limitations with lack of testing regarding quantitative accuracy and bias caused by deep learning. Extensive evaluation in phantoms was beyond this study, this decision was influenced by the fact that DL models are trained on human data, and simple phantoms might not accurately represent what is encountered in clinical practice. For the purposes of this study, the images were compared to our gold standard, ToF BSREM which has well characterized quantitative performance shown in previous publications [13]. The aim of this study was to demonstrate the potential of the sequential network application with comparison to ToF-BSREM images. The natural progression from this initial study would be to develop and train a network that incorporates aspects of DLE and DLT, at which point it would be feasible (and necessary) to test for quantitative accuracy (for example through inserted lesions) and biases.

This study demonstrated the potential for the sequential application of DLE and DLT to FDG scans. We aimed to explore this possibility and to openly identify any significant image artefacts or anomalies that might be produced. We did not find any notable such features in the images that were assessed; on the contrary, we found a reader preference for the combined use of the DL algorithms, and this was supported by several quantitative measures. From this single and moderate-sized study we certainly do not advocate the clinical use of the sequential application of the two DL algorithms. We do however consider this study may help uncover new directions for the use of Artificial Intelligence (AI) in PET. Specifically, if these two DL algorithms were able to provide image improvements when combined and used beyond their intended purpose, it is reasonable to postulate that a dedicated algorithm (either single- or two-stage) can be developed with an intended purpose similar to combining DLE and DLT and using similar architecture. Such an algorithm would be expected to outperform the sequential (mis-) use of DLE and DLT. The positive results obtained in this study thus motivate further work on the topic.

## Conclusion

The present study investigated the potential benefits of the sequential deployment of two deep learning algorithms: DLE and DLT. Our quantitative results show that, with ToF-OSEM as input, the sequential application of DLE and DLT can reduce noise in the liver and increase SUV_max_ of small target abnormalities significantly. This is reflected in the clinical reading where ToF-OSEM + DLE + DLT scored highest or second highest for lesion detectability and diagnostic confidence. The deployment of DLE may allow for faster scans and more rapid reconstruction times which could be of particular use for larger axial field of view cameras. Insight from this investigation can help guide future developments of AI and its use in PET.

### Electronic supplementary material

Below is the link to the electronic supplementary material.

Supplementary Material 1

## Data Availability

Data is available under reasonable request to the corresponding author.
