# Supplementary material for: Sequential deep learning image enhancement models improve diagnostic confidence, lesion detectability, and image reconstruction time in PET
Source: EJNMMI Phys. 2024 Mar 15;11:28. doi: 10.1186/s40658-024-00632-4 (PMC10942956; doi:10.1186/s40658-024-00632-4)
Supplement: Supplementary file 1 — Supplementary Material 1 [file 40658_2024_632_MOESM1_ESM.docx]

Table S1: Reconstruction times for one bed position for each type of reconstruction for D710 patients using a GE research console. Whilst reconstruction 1 is a GE HealthCare product the other reconstructions are research only and not products. Mean and standard error of the mean are shown across the patients with p-values calculated using a single tailed t-test relative to the time for reconstruction 1 in Python 3.11.

| Reconstruction number | Reconstruction and DL name | Time (s) |
| --- | --- | --- |
| 1 | ToF-BSREM | 208.7 ± 9.2 |
| 2 | ToF-OSEM + DLE | 101.0 ± 4.7 (p<0.0001) |
| 3 | OSEM + DLE + DLT | 86.9 ± 4.3 (p<0.0001) |
| 4 | ToF-OSEM + DLE + DLT | 125.1 ± 6.0 (p<0.0001) |
| 5 | ToF-BSREM + DLT | 232.8 ± 10.4 (p=0.005) |


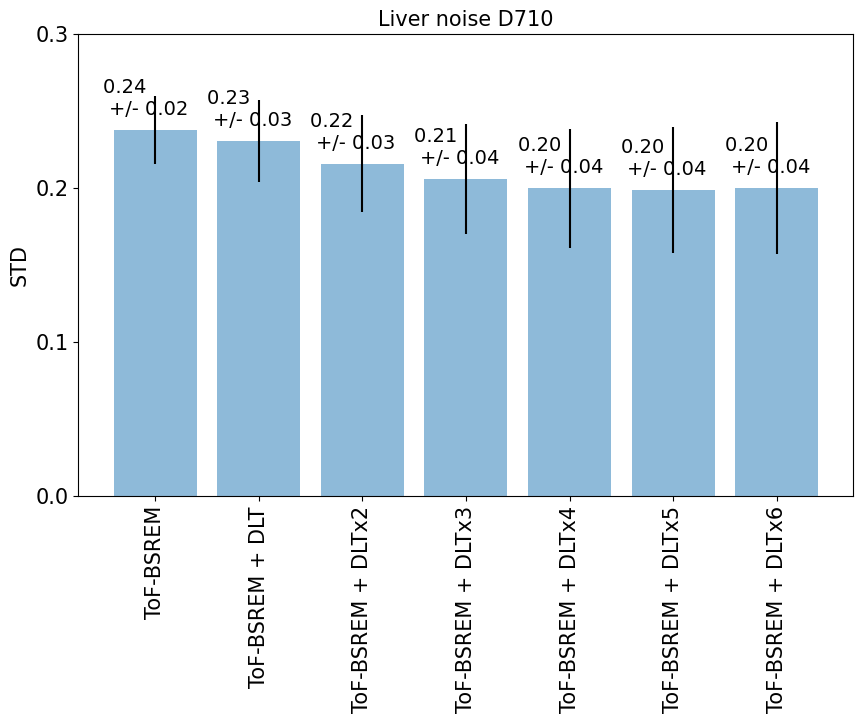

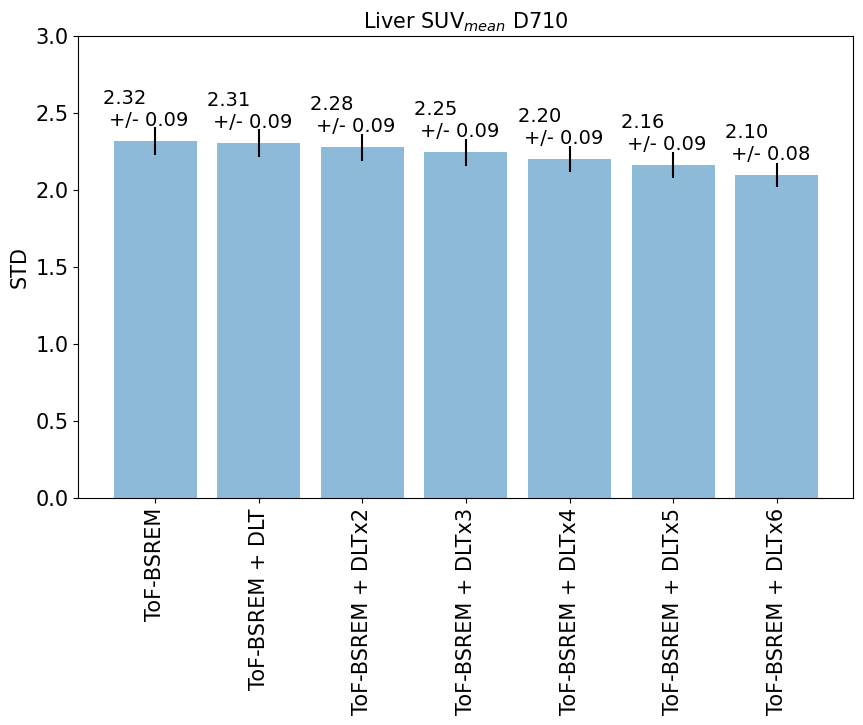

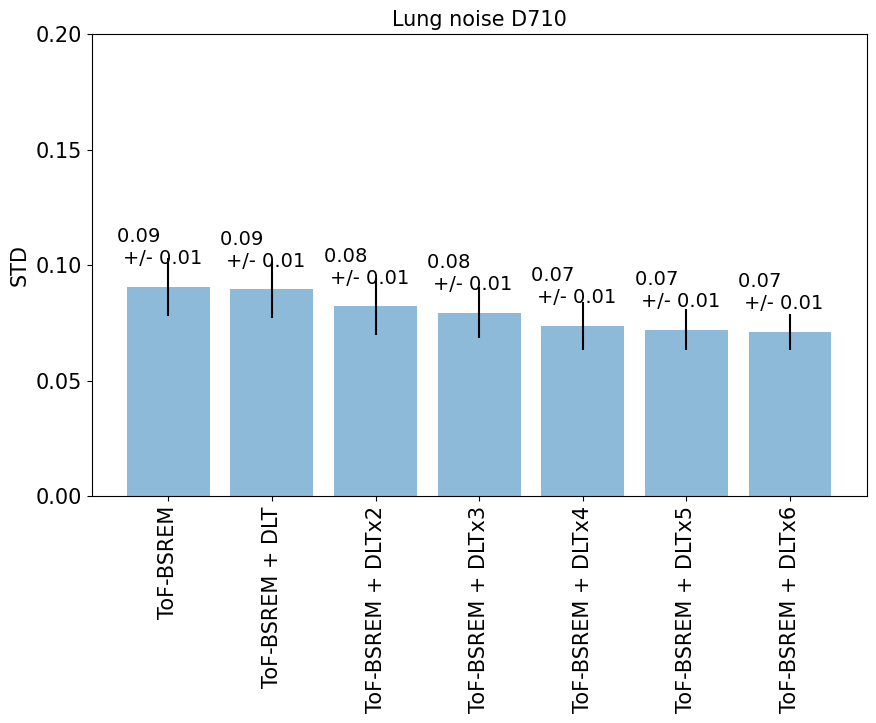

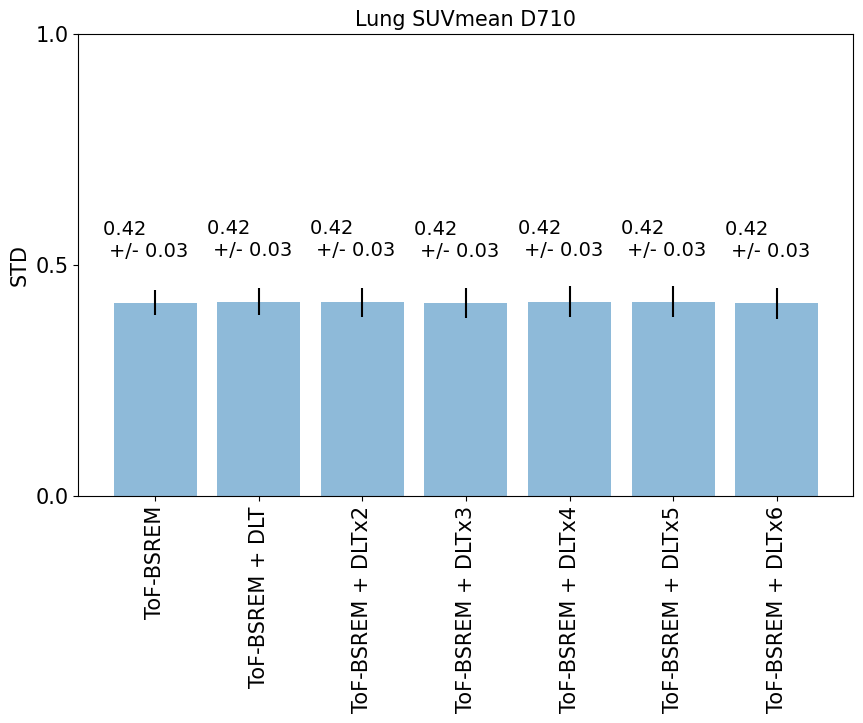


Figure S1: Quantitative performance of six sequential applications of DLT to ToF-BSREM images from D710 patient dataset. Tested by drawing a 30mm spherical VOI in both the lung and liver and measuring standard deviation between voxels and SUV_mean_ for 20 patients scanned on a GE HealthCare Discovery 710 scanner.


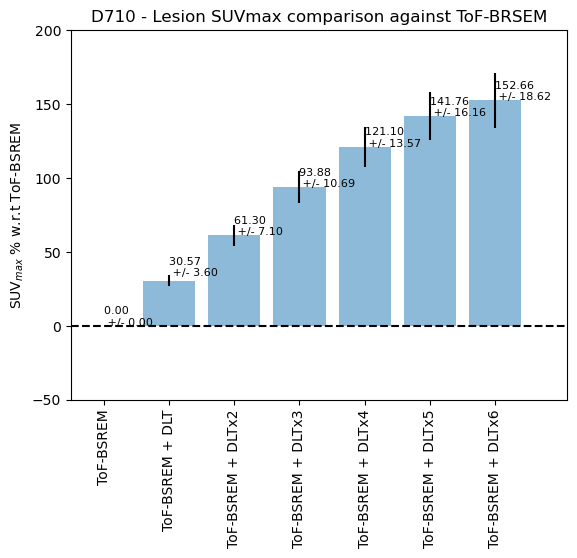


Figure S2: Quantitative performance of six sequential applications of DLT to ToF-BSREM images on lesion SUV_max_ scanned on a D710 PET-CT scanner. All lesions chosen were extreme cases where lesions were small (sub-cm)


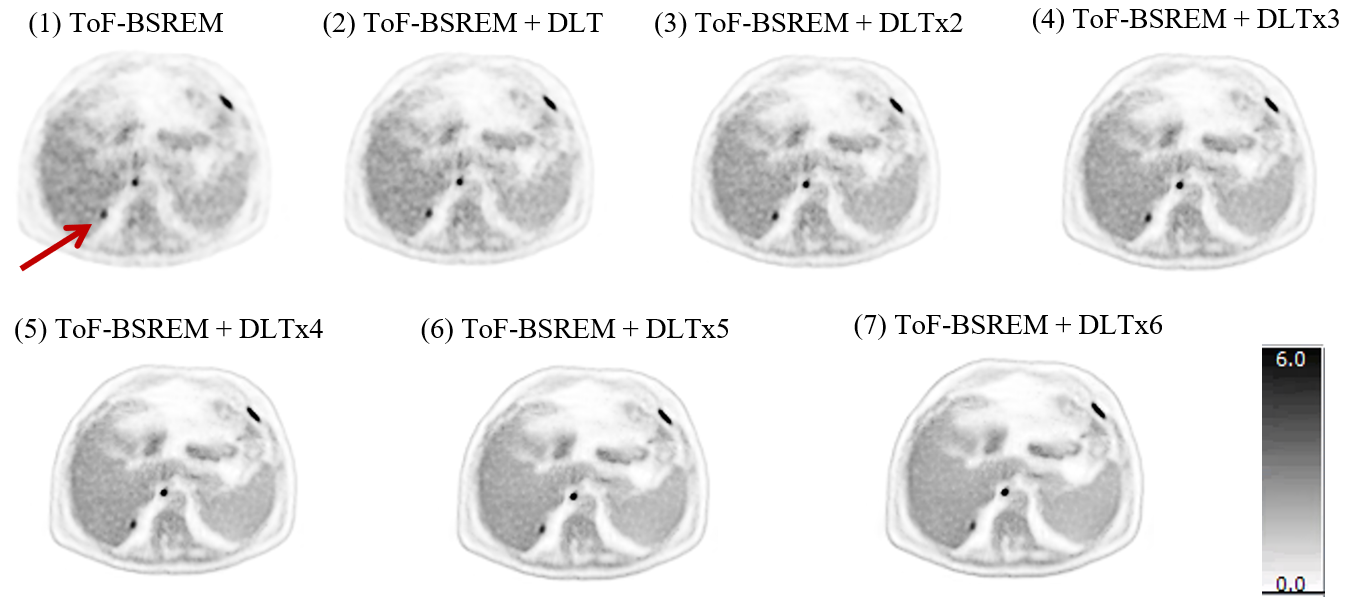


*Figure S3: Sequential Deep Learning Time-of-flight (DLT) applications to a test subject with BMI 19.4 kg/m^2^, with an injected activity of 229 MBq FDG, scanned on a D710 PET-CT scanner. The subject is a male patient, staging scan for relapsed, high grade, transformed, follicular, non-Hodkin’s lymphoma, with nodal and peritoneal disease. Axial PET images of a tiny peritoneal nodule posterior to the right lobe of the liver (red arrow) are demonstrated. All images use an SUV scale of 0-6.*
